# Supplementary material for: HER2-Low Status Is Not Accurate in Breast Cancer Core Needle Biopsy Samples: An Analysis of 5610 Consecutive Patients
Source: Cancers (Basel). 2022 Dec 15;14(24):6200. doi: 10.3390/cancers14246200 (PMC9777154; doi:10.3390/cancers14246200)
Supplement: Supplementary file 1 [file cancers-14-06200-s001.zip › cancers-2062362-supplementary.pdf]

Table S1. Concordance rate of molecular subtype between CNB and SES lesions.

CNB lesion	SES lesion					Concordance rate	Kappa	*P* value
	Luminal-A	Luminal-B/HER2-	Luminal-B/HER2+	HER2-amplified	TNBC			
Luminal-A	787	474	13	1	0	81.52%	0.750	**<0.001**
Luminal-B/HER2-	237	1882	41	0	62			
Luminal-B/HER2+	5	17	630	74	0			
HER2-amplified	0	1	44	561	3			
TNBC	1	46	3	14	714			

Abbreviations: CNB, core needle biopsy; SES, surgical excision specimen; ER, estrogen receptor; PR, progesterone receptor; HER2, human epidermal growth factor receptor-2; TNBC, triple negative breast cancer.

Table S2. Clinicopathological features according to HER2 discordance in HER2-Negative patients.

Characteristics	HER2 concordance, % N=3286	HER2 discordance, % N=989	*p* value
Age, years			0.380
< 55	1422 (43.3)	444 (44.9)	
≥ 55	1864 (56.7)	545 (55.1)	
BMI, kg/m^2			0.738
< 24	2000 (60.9)	596 (60.3)	
≥ 24	1286 (39.1)	393 (39.7)	
Menstruation			0.151
Pre/peri-menopausal	1163 (35.7)	376 (38.2)	
Post-menopausal	2099 (64.3)	608 (61.8)	
Time to surgery			0.177
< 1 week	2902 (88.3)	857 (86.7)	
≥ 1 week	376 (11.4)	129 (13.0)	
NA [a]	8 (0.2)	3 (0.3)	
Histology [*]			0.097
IDC	2866 (87.2)	842 (85.1)	
Non-IDC	420 (12.8)	147 (14.9)	
Grade [*]			0.052
I	154 (4.7)	51 (5.7)	
II	1807 (55.0)	499 (55.4)	
III	1050 (32.0)	350 (38.9)	
NA [a]	275 (8.4)	89 (9.0)	
Tumor size, cm			0.136
≤ 2	1765 (53.7)	504 (51.0)	
> 2	1519 (46.2)	484 (48.9)	
NA [a]	2 (0.1)	1 (0.1)	
Nodal status			0.475
Negative	2052 (62.6)	631 (63.9)	
Positive	1225 (37.4)	356 (36.1)	
NA [a]	9 (0.3)	2 (0.2)	
ER [#]			<0.001
Positive	2715 (82.6)	762 (77.0)	
Negative	571 (17.4)	227 (23.0)	
PR [#]			<0.001

Positive	2347 (71.4)	646 (65.3)	
Negative	939 (28.6)	343 (34.7)	
Ki67 [#], %			<0.001
< 20	1662 (50.6)	422 (42.7)	
≥ 20	1624 (49.4)	567 (57.3)	
Molecular subtype [#]			<0.001
Luminal-A	1016 (30.9)	259 (26.2)	
Luminal-B/HER2-Negative	1715 (52.2)	507 (51.3)	
TNBC	555 (16.9)	223 (22.5)	

[a] NA patients are not included in statistical analysis.

[#] IHC categories are based on core needle biopsy samples.

[*] Histological and pathological categories are based on surgical excision samples.

Abbreviations: CNB, core needle biopsy; HER2, human epidermal growth factor receptor-2; BMI, body mass index; IDC, invasive ductal carcinoma; NA, not available; ER, estrogen receptor; PR, progesterone receptor; IHC, immunohistochemistry; TNBC, triple negative breast cancer

Supplementary Table S3. Main researches about accuracy of core needle biopsy cited in the text.

Study	Type	Biomarker	Accuracy
Shanmugalingam A, et al. [11]<br>Australia (2022), N=504	Retrospective	ER, PR, HER2(-/+), Ki67, Grade	ER 96.7%, PR 93.2%, HER2 100%, Ki67 72.6%, Grade 70.5%
Chen X, et al. [13]<br>China (2013), N=298	Retrospective	ER, PR, HER2(-/+), Ki67, Molecular subtype	ER 93.6%, PR 85.9%, HER2 96.3%, Ki67 77.2%
Zhu S, et al.[16]<br>China (2019), N=1710	Retrospective	HR (=ER+PR)	HR 96.5%
Miglietta F, et al. [18]<br>Italy (2022), N=446	Retrospective	HER2(-/low/+) after NAC	HER2 73.6%
Chen X, et al. [12]<br>China (2012), 27 studies	Meta-analysis	ER, PR, HER2(-/+)	Sensitivity: ER 0.970, PR 0.911, HER2 0.799<br>Specificity: ER 0.790, PR 0.730, HER2 0.890
Kalvala J, et al. [31]<br>United Kindom(2022), 22 studies	Meta-analysis	Ki67	Ki67 70.3-92.7%
